# A Computer Model of Starve Fed Single Screw Extrusion of Wood Plastic Composites

**DOI:** 10.3390/polym13081252

**Published:** 2021-04-12

**Authors:** Krzysztof J. Wilczyński, Kamila Buziak

**Affiliations:** 1 Politech Ltd., 86-031 Osielsko, Poland; 2 Polymer Processing Department, Faculty of Production Engineering, Warsaw University of Technology, 85 Narbutta, 02-524 Warsaw, Poland; kamila.buziak@pw.edu.pl

**Keywords:** computer modeling, starve fed extrusion, wood plastic composites

## Abstract

In this study, we present a computer model of starve fed single screw extrusion of wood plastic composites (WPC). Experimental studies have been performed on the extrusion of the polypropylene (PP) based composites with various wood fiber contents (WF). The melting mechanisms of the composites in the extruder have been observed, and melting models have been proposed for partially and fully filled sections of the screw. It was observed that in the partially filled section the material is melted by conduction, as in the case of extrusion of neat polymers. On the other hand, in the fully filled section, the Tadmor melting mechanism appears, which is different compared to the melting mechanism of neat polymers at starve fed extrusion, where dispersed melting is observed. Using the melting models, the global computer model of the process has been developed which makes it possible to predict the process flow, i.e., the polymer melt temperature and pressure, the polymer melting rate, and the degree of screw filling. To build the model, the specific forward/backward procedure was developed, which consists in determining “forward” the melting profile, and “backward” the pressure and screw filling profile. The temperature profile in the melting section is computed “forward”, while “backward” in the metering section. This procedure makes it possible to solve the crucial problem of modeling of the starve fed extrusion process, which is to find the location of the point where the screw is fully filled, and the pressure is developed. The model has been tested by pressure measurements in the extruder.

## 1. Introduction

Extrusion can be performed using two ways of feeding. The conventional feeding by gravity, often called as flood feeding, is mostly applied in single screw extruders. The non-conventional, metered feeding, also known as starve feeding, is primarily applied in twin screw extruders. Extrusion with metered feeding ensures faster melting and better mixing of the material, and therefore, this may be also applied in single screw extruders.

**Figure 1:**
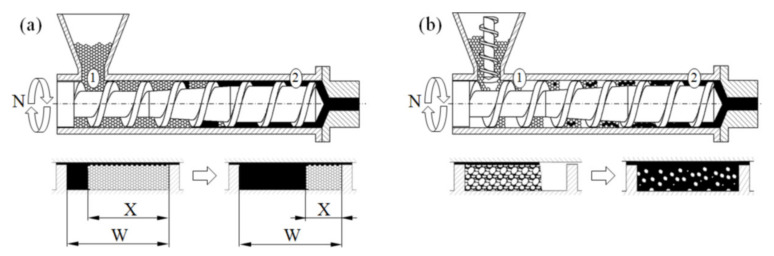
Mechanisms of melting (fusion) for single screw extrusion: (**a**) flood fed extrusion, (**b**) starve fed. extrusion, 1—solid conveying section, 2—melt conveying section, X—solid bed width, W—screw channel width [1].

In the case of feeding by gravity, the screw takes as much polymer as it can, and the screw is completely filled with it. In the case of metered feeding, the polymer is delivered to the extruder by a dosing device, and the beginning section of the screw is only partially filled with the polymer. This is shown in Figure 1.

The starve fed extrusion has several advantages over the flood fed extrusion, as discussed in the literature, e.g., [2,3,4,5,6,7], and very recently in [8]. The pressure development in the extruder is lower, the polymer does not agglomerate, and mixing is substantially better. Polymer fusion (melting) is faster since the granules are not compacted into a solid bed, and retain their separateness during fusing. In the case of metered feeding, the rotational speed of the screw can be changed at the constant extruder throughput, and the extruder throughput can be changed at the constant rotational speed of the screw, which enables for better process control. In general, the extruders with metered feeding may be applied to perform the more advanced processing tasks, e.g., [8], although this way of feeding also has some drawbacks. The extrusion throughput is lower, and the process is more complicated since an additional device is used to deliver the polymer to the extruder.

The conventional single screw extrusion is relatively well understood, and well described in the literature. Tadmor et al. were the first who proposed the mathematical model of melting (fusion) of polymers in the single screw extruder, and later the global (composite) model of the process, which was composed of the models of solid conveying, fusion of the polymer, and melt conveying [9,10,11]. Later, several other models of polymer fusion were built, and several composite computer models of conventional single screw extrusion were proposed [12,13,14,15]. The classical mechanism of fusion of polymers in the conventional, flood fed single screw extruder is depicted in Figure 1a.

The state-of-the-art of modeling the extrusion process was discussed in the fundamental monographs, e.g., of Rauwendaal [7], White and Potente [16], Tadmor and Gogos [17], Osswald and Hernandez-Ortiz [18], Agassant et al. [19], and in some research papers, e.g., [20,21,22,23,24]. Wilczyński et al. summarized this in the review paper [25].

Although the conventional extrusion was widely studied and described in the literature, little information was delivered on the extrusion with metered feeding. Wilczyński et al. [26,27] performed extensive experimental studies of this process, and proposed the mechanism and mathematical model of polymer fusion, and then built the first composite computer model of this [28]. In the model, two steps of polymer fusion were distinguished; fusion by heat conduction in the partially filled section of the screw, and fusion by energy dissipation in the completely filled section of the screw. These first models were later extended to the extrusion with the use of non-conventional screws [1,29], and to the extrusion of polyblends [30,31]. The mechanism of fusion of polymers in the single screw extruder with metered feeding is depicted in Figure 1b.

Wood plastic composites (WPC) are composed of the thermoplastic matrix and the wood filler, that is, the wood flour or fiber. These materials have high durability, high stiffness/strength and low price compared to other competing materials. In addition, they are water and weather resistant and can be used for a variety of outdoor applications.

The wood plastic composites (WPC) market has grown substantially in the last years, which primarily results from increasing the building industry and the automotive industry. The most used wood plastics composites are polypropylene (PP) composites, high density polyethylene (HDPE) composites, and polyvinyl chloride (PVC) composites [32,33,34,35].

The fundamental technology for processing wood plastic composites (WPC) is extrusion due to an extensive use of these as profiles. Extrusion of wood plastic composites differs substantially from extrusion of thermoplastics, which results from different thermo-rheological characteristics of these filled materials, various structure, etc.

Limited studies were performed on rheology of wood plastic composites (WPC), as well as on extrusion of these. Basic research was carried out by Xiao and Tzoganakis [36,37,38,39] and by Li and Wolcott [40,41,42]. Valuable contributions to the issue were also made by Oksman Niska and Sain [33], Vlachopoulos et al. [43,44,45], and Zolfaghari et al. [46].

Currently, it is generally known that wood plastic composites (WPC) are pseudoplastics and viscoelastics. Their viscosity lowers when the shear rate and temperature rise and rises when the filler content rises.

Wood plastic composites (WPC) exhibit slipping and yield stress, which was presented and discussed by Xiao and Tzoganakis [36], Li and Wolcott [40], and Hristov et al. [43]. It was observed that the slip velocity depends on the wood filler content and shear rate. It rises when the shear rate rises, which may lead to the plug flow as reported by Vlachopoulos and Hristov [44]. In addition, increasing the wood filler content may promote the plug flow as reported by Zolfaghari et al. [46].

Designing an extrusion process of wood plastic composites (WPC) requires a deep knowledge on the flow mechanism of these in the extruder. Only a few fusion studies were carried out, e.g., by Xiao and Tzoganakis [36,37,39] for a high-density polyethylene (HDPE) composite. The experimental results of these studies were compared to simulations performed by using a commercial (unknown) software. However, the computations of pressure were not consistent with those measured.

Wilczyński et al. [47] performed extensive experimental research on extrusion of the polypropylene (PP) based wood plastic composites (WPC), which have shown that the flow and fusion of these materials are considerably different from those of the neat polypropylene (PP). These studies were performed using a conventional process with gravitational feeding, i.e., flood feeding. It was concluded that the solid conveying and material fusion were dependent on the material composition and process operating conditions. The contiguous solids melting (CSM), which is known as a Tadmor mechanism [10], was not observed for composites with higher wood filler (WF) content. However, it was seen for composites with lower (WF) content, with up to 50%. Thus, the composite fusion is dependent on the wood filler content (WF), and for the lower (WF) content the classical 2D fusion takes place, while for the higher (WF) content 1D fusion is seen. In the classical 2D model, when fusion progresses, the volume of the solid material diminishes by diminishing the solid width, while in the 1D model, the volume of the solid material diminishes by diminishing the solid height. In this case, increasing the melt volume, i.e., increasing the melt film thickness, diminishes the energy dissipation since the shear rate lowers when the film thickness rises, and this causes slower melting.

➢ Based on these investigations, the first global (composite) computer model of single screw extrusion of wood plastic composites (WPC) has been developed for extrusion with flood feeding. The model predicts the extrusion throughput, the polymer melting temperature and pressure, the melting (fusion) rate, and the power consumption. The model has been successfully tested by experiment [35].
➢ Up to now, there is not any model of starve fed single screw extrusion of wood plastic composites (WPC). The only preliminary simulation studies were performed for this process using the classical computer model of starve fed extrusion developed for thermoplastics [48].

In the research presented here, experimental studies have been performed on the extrusion of polypropylene (PP) composites with various wood fiber contents (WF), and a novel global computer model of single screw extrusion of wood plastic composites (WPC) with metered feeding has been developed.

## 2. Experimental

In the study, a single screw extruder was used with a screw/barrel system of a diameter D = 45 mm and a length/diameter ratio (L/D) = 27. The conventional screw was applied with the section of feeding (L/D)_F_ = 10.78, the section of compression (L/D)_C_ = 7.11, and the section of metering (L/D)_M_ = 9.11. The channel depth (H_F_) in the feeding section was equal to H_F_ = 8 mm, and the channel depth (H_M_) in the metering section was equal to H_M_ = 3 mm.

A flat die for tapes of width W = 20 mm and thickness H = 2 mm was applied. A “screw pulling-out technique” was used to analyze the material transport and fusion mechanism. This well-known technique is described elsewhere [31]. The pressure was measured along the screw/barrel system, and in the die. The screw speed was equal to *n* = 30 rpm, *n* = 70 rpm, and the barrel/die temperatures were equal to T_I_ = 150 °C, T_II_ = 175 °C, T_III_ = 180 °C, T_IV_ = 180 °C, and T_DIE_ = 180 °C. The extrusion process was performed at the flood feeding, and at the starve feeding with the flow rates equal to G_SF 1_ = 0.9 G_FF_, and G_SF 2_ = 0.8 G_FF_, where G_FF_ is the flow rate at the flood feeding, which each time is dependent on the process and material conditions.

The polypropylene (PP) based composites were used in the study. These were (WPC) of compositions (WF/PP) equal to 20/80 and 40/80, i.e., (i) WPC 20/80 ForMi GP20 and (ii) WPC 40/60 ForMi EXP40 (manufactured by The Biofore Company UPM, Helsinki, Finland).

In is worth noting here that in terms of product segmentation, the (WPC) market is categorized into polyethylene, polypropylene, and polyvinylchloride [34]. The polypropylene segment is anticipated to see a boom, owing to its heavy use in niche application segments such as water-resistant coatings on furniture and high temperature controllable wooden units.

The composites used in the study are cellulose fiber reinforced plastic composites with high renewable material content. Principal ingredients are specially selected cellulose fibers and virgin polypropylene. Cellulose fibers significantly increase the stiffness and strength of polypropylene. These environmentally sound composites are optimized for use in extrusion, and injection molding applications instead of polypropylene, filled polypropylene, or several other plastics. These are fully recyclable or can be burned for energy. All cellulose fibers are from sustainably managed forests.

A small amount of the composite (4%) has been colored with 2% of Ramafin Orange CEK071 dye-stuff to distinguish between the solid and melt. The material was dried at 80 °C for 4–6 h.

The (WPC) composites (20/80 and 40/60) have, respectively, the solid density equal to ρ_s_ = 0.99 g/cm^3^ and ρ_s_ = 1.08 g/cm^3^, the melt density equal to ρ_m_ = 0.85 g/cm^3^ and ρ_m_ = 0.98 g/cm^3^ at the temperature 190 °C, and the bulk density equal to ρ_bulk_ = 0.4–0.6 g/cm^3^. The melting point is about T_m_ = 160 °C.

**Figure 2:**
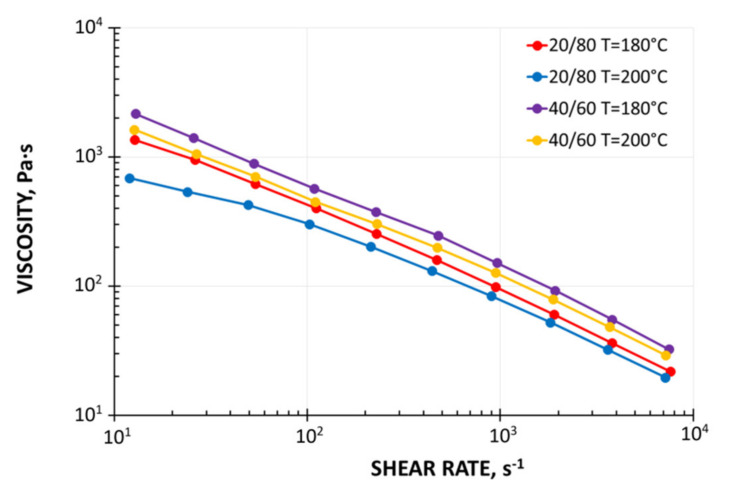
Viscosity curves of WPC composites: WPC 20/80 and WPC 40/60.

Viscosity curves were determined for both composites. The tests were performed with the use of capillary rheometer RG-25 (from Goettfert, Buchen, Germany). The four capillaries of diameter D = 1 mm, D = 2 mm, and the length/diameter ratios L/D = 0/1, 0/2, 10/1 and 20/2 were used, and the Rabinowitsch and Bagley corrections were applied in rheological computations. The measurements were performed in the temperature ranges of 180 °C, 190 °C, and 200 °C and in the shear rate range of 1–2000 s^−1^. The viscosity curves are presented in Figure 2, and these show a typical result for pseudoplastics decreasing the viscosity with increasing the shear rate. However, the viscosity depends only slightly on the temperature.

A mass flow rate (MFR) index was determined for both composites with the use of Melt Indexer MI-2 (from Goettfert, Buchen, Germany). The tests were made at the temperature 190 °C, and two loads were applied, the Low Load (LL = 5.0 kg) and the High Load (HL = 21.6 kg), and the (MFR) indexes were equal to MFR_LL_ = 17 g/10 min., MFR_HL_ = 580 g/10 min. for WPC 20/80, and MFR_LL_ = 3 g/10 min., MFR_HL_ = 92 g/10 min. for WPC 40/60.

## 3. Results

The goal of experimentation was to learn the flow mechanism of composites in the extruder, as well as the fusion mechanism. These were the starting points for extrusion modeling, as well as for the validation of the model developed.

**Figure 3:**
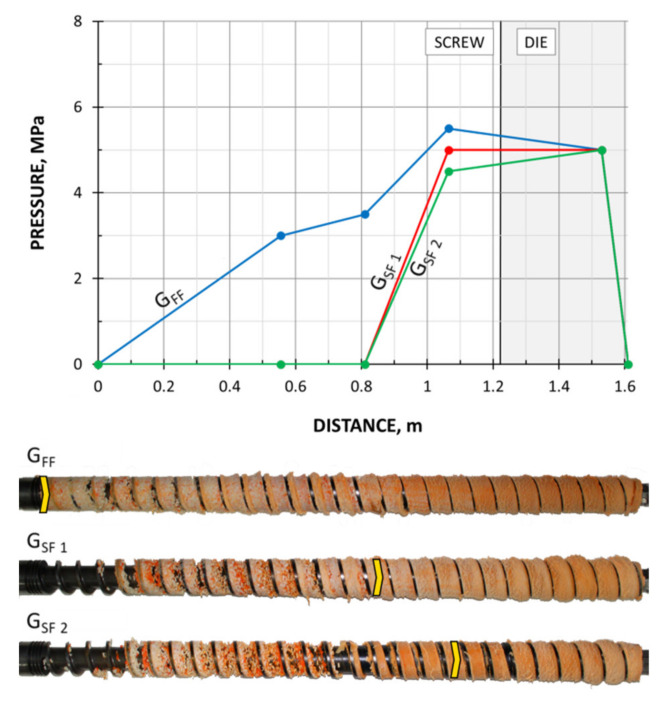
Experimental data for WPC of WF/PP = 20/80, *n* = 30 rpm (at flood/metered feeding): pressure, data, and screws pulled out of the extruder (the arrow indicates the beginning of the fully filled section).

**Figure 4:**
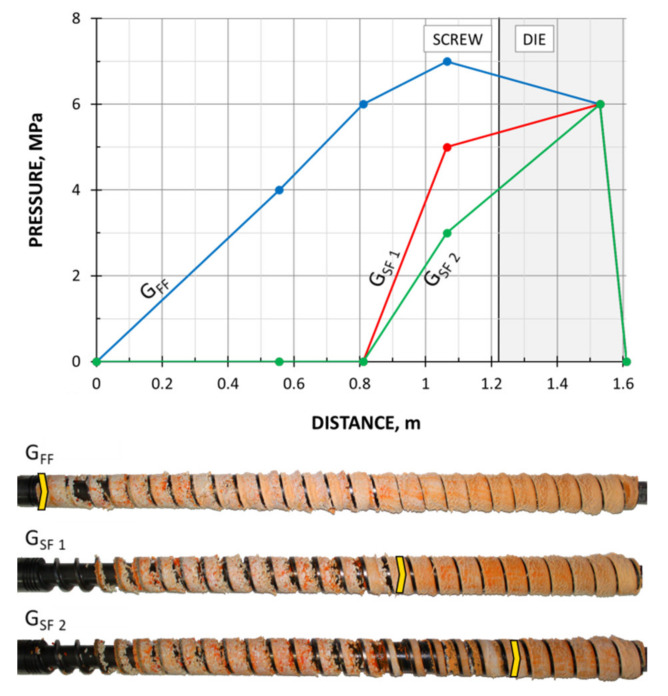
Experimental data for WPC of WF/PP = 20/80, *n* = 70 rpm (at flood/metered feeding): pressure, data, and screws pulled out of the extruder (the arrow indicates the beginning of the fully filled section).

**Figure 5:**
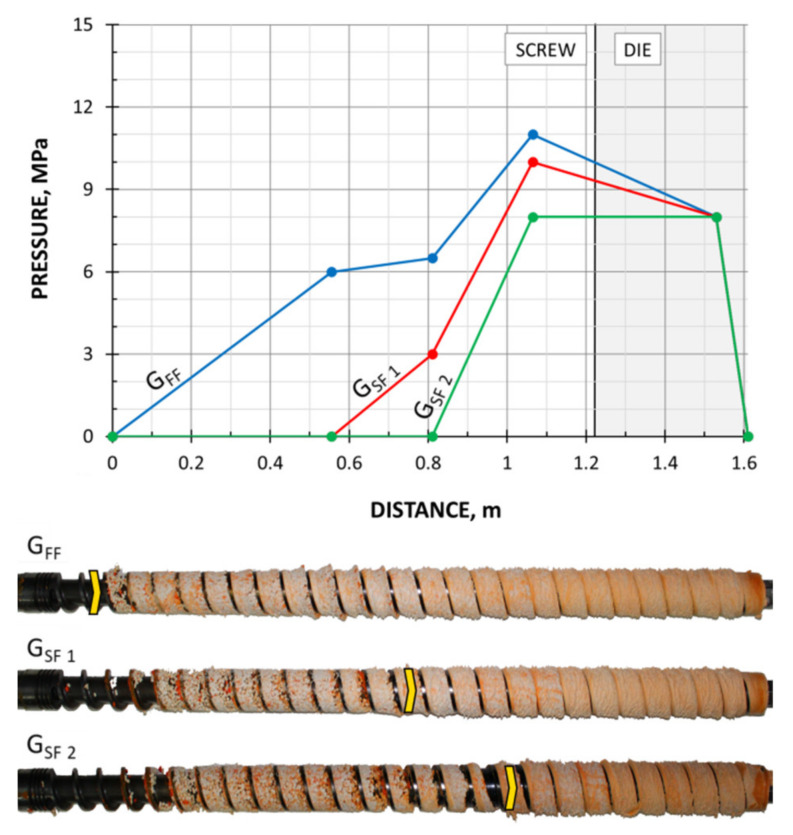
Experimental data for WPC of WF/PP = 40/60, *n* = 30 rpm (at flood/metered feeding): pressure, data, and screws pulled out of the extruder (the arrow indicates the beginning of the fully filled section).

**Figure 6:**
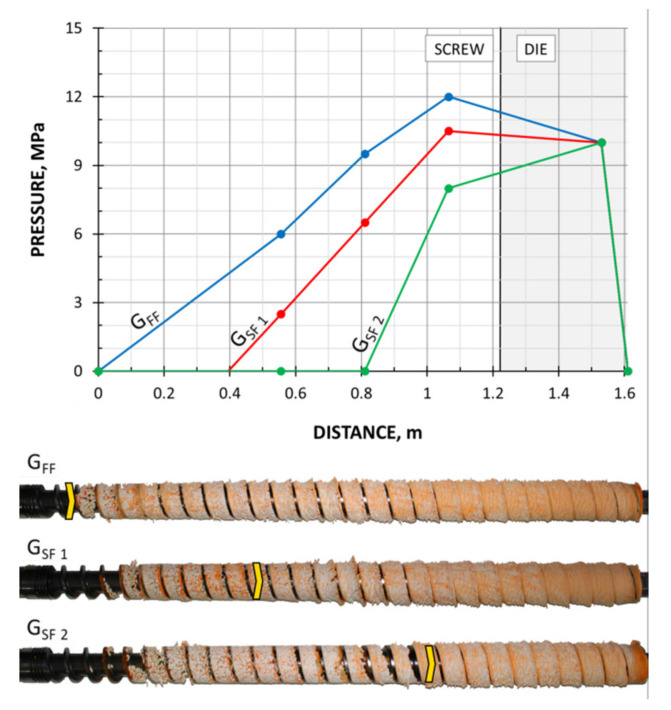
Experimental data for WPC of WF/PP = 40/60, *n* = 70 rpm (at flood/metered feeding): pressure, data, and screws pulled out of the extruder (the arrow indicates the beginning of the fully filled section).

The screws, which were pulled out of the extruder, and the results of pressure measurements are presented in Figure 3, Figure 4, Figure 5 and Figure 6. At the flood fed extrusion, the screws are fully filled with the material, and the pressure is developed over the entire length of the screw. At the starve fed extrusion, the screws are not fully filled in their initial part, and the pressure is not developed here. The screws fill up in their second part, and the pressure is developed here (the arrows indicate the beginning of the fully filled section of the screw). When flood feeding, the pressure developed is higher than at starve fed extrusion. When using metered feeding, the pressure developed is higher when the flow rate is higher (G_SF 1_ > G_SF 2_). The length of the fully filled section of the screw is also higher when the flow rate is higher (G_SF 1_ > G_SF 2_).

**Figure 7:**
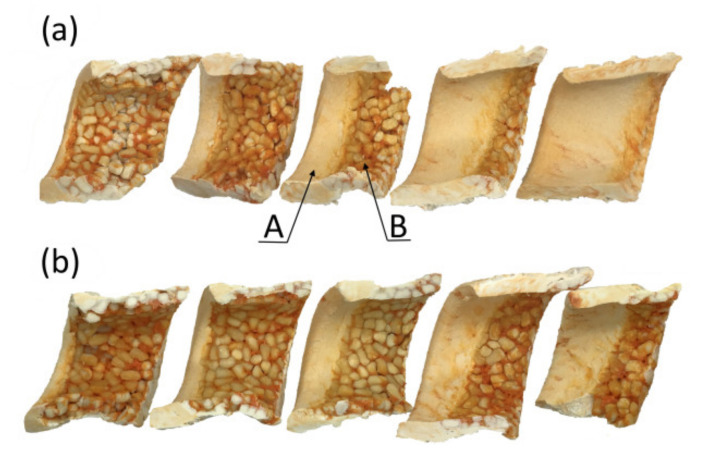
An effect of the screw speed on the material flow for extrusion of WPC of WF/PP = 40/60 (at flood feeding, samples of the material removed from the screw, a view from the screw side): A—melt, B—solid, (**a**) *n* = 30 rpm, (**b**) *n* = 70 rpm.

Examples of the material samples removed from the screw are depicted in Figure 7. When flood feeding, it is seen that with an increase of the screw speed, the fusion starts further away from the hopper, and closer to the die. Fusion seems to be slower as the residence time of the material in the extruder is shorter.

**Figure 8:**
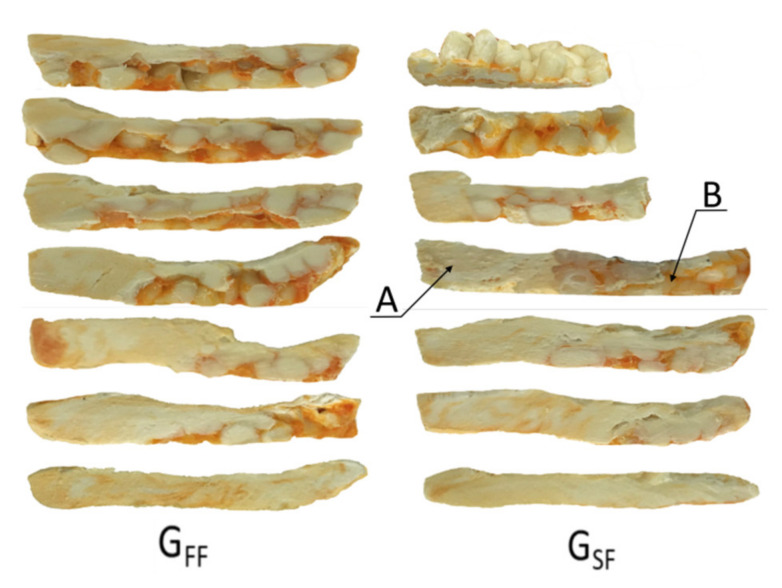
An effect of the feeding mode on the material flow for extrusion of WPC of WF/PP = 40/60 (at flood/metered feeding, G_FF_/G_SF_): A—melt, B—solid.

Examples of the cross-sections of the material samples removed from the screw are depicted in Figure 8. When flood feeding (G_FF_), the classical 2D fusion is seen, while when metered feeding (G_SF_), the fusion by conduction is seen in the partially filled section of the screw, and the classical fusion is observed in the fully filled section which is different compared to the fusion mechanism of neat polymers at the starve fed extrusion, where dispersed fusion is observed [26].

**Figure 9:**
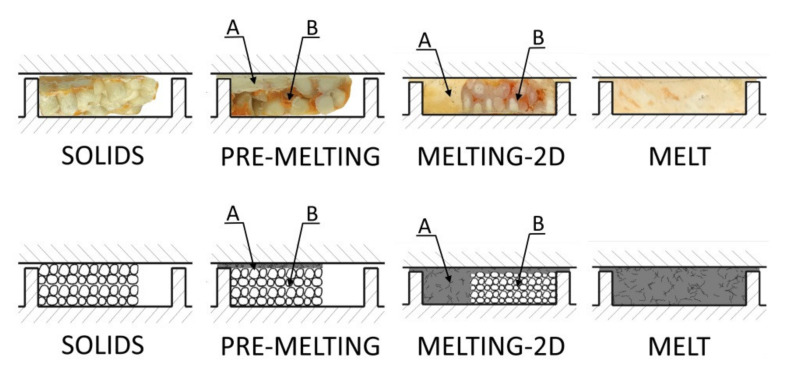
Fusion mechanism of wood plastic composite (WPC) in starve fed extrusion: visualization of fusion and schematic model of fusion, A—melt, B—solid.

Based on the experimental observations, melting (fusion) mechanisms for starve fed extrusion of wood plastic composites (WPC) have been proposed, which are depicted in Figure 9. In the partially filled section, fusion by conduction was observed, while in the fully filled region, the classical Tadmor mechanism was seen.

It was seen that at the beginning of the screw, the material thickens gradually, starting from the active flight of the screw. It is here melted in the whole mass mainly by conduction, as in the case of extrusion of neat polymers, e.g., low density polyethylene (LDPE), polypropylene (PP), and polystyrene (PS), as reported in [25]. On the other hand, in the area of the fully filled screw, the classical Tadmor fusion mechanism appears with a melt pool at the active flight of the screw, which is different comparing to the fusion mechanism of these polymers at the starve fed extrusion. Fusion begins at the contact surface between the material and the barrel, where a thin film of the molten material is formed. Then this molten material is scrapped off towards the active flight of the screw, where the melt pool is formed. As fusion proceeds, the melt pool grows, so that the fusion progresses across the screw channel.

## 4. Modeling

Modeling of the extrusion process must include both the flow in the screw and the flow in the die, and must take into consideration the interaction between these, describing the flow phenomena in the screw/die system.

The flow of polymers through extrusion dies is rather well understood and mathematically modeled for viscous flows. However, this is not for viscoelastic flows, as well as for materials such as wood plastic composites (WPC), for the reason of possible slipping at the wall and yield stress. The flow of polymers in extruders (screws) is much more complex, as it involves the solids conveying, the polymer fusion, and the melt conveying. This is difficult to model even in the case of neat polymers for viscous flows, and extremely difficult and still poorly understood in the case of complex materials such as wood plastic composites (WPC).

In the classical extrusion (with gravitational feeding), the mass/volume flow rate of the polymer is not a known a-priori since it is not set by the process operator, but it is due to the co-operation of the screw/die system. Process conditions of this co-operation are defined by the extruder operating point, which determines the extruder throughput (the polymer flow rate), and the extruder die pressure. In this case, the fundamental for the computation procedure is solving the problem of simultaneously computing the flow rate and the pressure in the screw/die system. This can be obtained by iterative computations in which the pressure increase in the screw is compared with the pressure drop in the die, and the convergence of these computations is sought This classical modeling approach was presented, e.g., in [49].

In the extrusion process with metered feeding, the flow rate of the polymer is known as it is set by the process operator. When process modeling, there is no need to search for the extruder operating point. However, it is not known here where the screw is completely filled with the material. This can be computed and defined by iterative computations in which the computed polymer melt temperature is compared with the polymer fusion temperature at the point where the fusion finishes. This modeling approach was presented, e.g., in [28].

The composite extrusion model may be represented as a series of connections of the elementary models that describe the solid conveying, the polymer fusion, and the melt conveying, as well as the flow in the die.

For engineering computation purposes, the lumped parameter approach is sufficient to apply [17]. According to this, the separate elementary process increments may be considered with the locally constant process/material parameters. These parameters, e.g., temperature, pressure, solid/melt volume, at the exit of each increment are equal to those at the beginning of the next increment, i.e.,

f_i_out_ (z) = f_i + 1_in_ (z + Δz), (1)

where f_i_out_ (z) is the process parameter (e.g., temperature, pressure, solid/melt volume at the end of i–increment, f_i + 1_in_ (z + Δz) is the process parameter at the beginning of (i + 1)–increment, z is the location of the increment, and Δz is the length of the screw channel.

The global (composite) extrusion model, which is discussed here, contains certain elements of our previous models [28], and introduces new algorithms and new concepts for fusion, which result from the studies presented here.

Summarizing the model consists of the following elementary models:

(1) the partially filled region: fusion by conduction.
(2) the fully filled region: fusion according to the classical 2D model instead of the dispersed fusion model, which is valid for neat polymers.
(3) the polymer melt conveying in the screw: modeling with the use of 3D FEM based screw pumping characteristics.
(4) the polymer melt flow in the die: modeling with the use of 1D approach.

**Figure 10:**
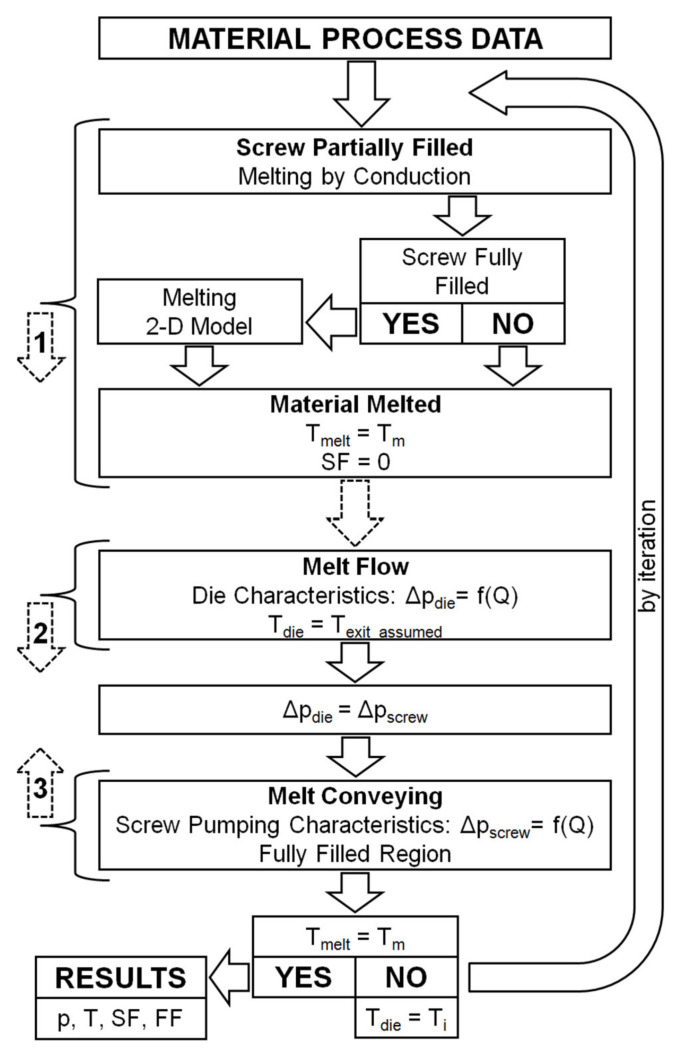
Scheme of computations: Q—flow rate, p—pressure, Δp_screw_—pressure increase in the screw, Δp_die_—pressure drop in the die, T—temperature, T_melt_—melt temperature, T_die_—die temperature, T_exit_assumed_—melt temperature at the screw end, in the first iteration, T_i_—melt temperature in the next iteration, T_m_—melting point (fusion temperature), SF—melting (fusion) profile (Solid Fraction), FF—screw filling profile (Fill Factor).

The scheme of computations has been depicted in Figure 10. These computations are complex and require a specific forward/backward procedure. This procedure consists in determining the fusion profile of the material in the extruder, from the hopper to the die, i.e., forward, and the pressure and screw filling profiles in the opposite direction, from the die to the hopper, i.e., backward. The temperature profile in the fusion section is determined in the forward direction, and in the backward direction in the metering section. This procedure makes it possible to locate the point where the screw is fully filled with the material. From this point, the pressure is developed, and the fusion mechanism is changed. The arrows in the Figure 10 represent the direction of computation, that is, the forward direction in the area of polymer fusion in the screw and in the area of polymer melt flow in the die, and the backward direction in the area of polymer melt flow in the screw.

In the initial part of the screw, in the partially filled screw section, the polymer fusion is modeled using the conductive model. In the fully filled screw section, the 2D model is used. These computations are performed in the forward direction. When fusion is completed, the computations are transferred towards the die. Starting with the initially assumed melt temperature, the pressure drop in the die is computed in the forward direction. From the principle, it is equal to the pressure increase in the screw. Then, the screw pumping characteristics is used, and the pressure gradient over the screw channel in the backward direction is computed. When pressure diminishes to zero, starving develops, and filling of the screw is computed. The process parameters are computed in the backward direction until the fusion is completed. At this point, the polymer melt temperature is compared with polymer fusion temperature. The computations are repeated by iterations of the modified die melt temperature, and the convergence of these computations is sought. At each iteration, the fusion computations are performed, and the fusion end as well as the melting profile are changed.

In the partially filled section, an energy balance is performed on the elementary material volume [27]. The pressure and the friction heat are not generated here, thus

Gc_s_ (dT/dz) = h_b_X_b_(T_b_ − T) + h_s_X_s_(T_s_ − T), (2)

where G is the polymer flow rate, c_s_ is the polymer solid specific heat, T is the polymer mean temperature; T_b_ and T_s_ are the temperatures of the barrel and the screw, respectively, h_b_ and h_s_ are the heat transfer coefficients at the barrel and screw surfaces, respectively, X_b_ and X_s_ are the widths of the polymer solid at the barrel and screw surfaces, respectively.

The polymer gets hot according to this equation, and the polymer temperature rises. When the polymer fusion temperature is reached, the fusion starts. The energy rate that is necessary to melt the polymer is expressed as

q_fusion_ = G[c_s_(T_m_ − T_0_) + λ], (3)

where q_fusion_ is the energy rate, which is necessary for polymer fusion, T_m_ is the polymer fusion temperature, T_0_ is the starting temperature, and λ is the fusion heat.

In the fully filled screw section, the classical 2D model is applied. The velocity and temperature distributions in the melt film and temperature distribution in the solid bed are determined first, and then an energy balance at the melt/solid interface and the mass balance in the melt film and the solid is made, which allows to predict the fusion rate, e.g., [49]. Following the energy balance, the heat flux used for the polymer fusion is equal to the difference between the heat flux from the melt film into the interface and the heat flux from the interface into the solid bed.

The composite modeling of the extrusion process requires iterative, multiple calculations (hundreds of iterations), and for that reason, the time-consuming finite element method (FEM) is not applied here. However, the pumping flow characteristics based on the (FEM) computations may be developed, and implemented in the form of regression models into the composite model. In this way, a good accuracy of calculations may be achieved within a reasonable time.

The screw pumping characteristics are the dimensionless functions of the flow rate and the pressure gradient

Q^x^ = f (Δp^x^), (4)

where Q^x^ is the flow rate (dimensionless), and Δp^x^ is the pressure gradient (dimensionless).

This modeling approach has been recently used by the authors for modeling the single screw extrusion with conventional screws [28] and with mixing screws [1,29], operating in both feeding modes; metered feeding and flood feeding.

In the study presented here, the single screw extrusion with the use of conventional screws is modeled. Thus, the screw pumping characteristics may be presented as [35]

(5) $$Q^{x}=\;\frac{2Q}{{\mathrm{WHV}}_{\mathrm{bz}}},$$

(6) $${\Delta p}^{x}=\;\frac{H^{n\;+\;1}\cos\varphi }{6{\mathrm{mV}}_{\mathrm{bz}}^{n}}\cdot \frac{{\Delta p}_{\_\mathrm{ch}}}{L_{p\_\mathrm{ch}}},$$

where Q is the volumetric flow rate, W is the width of the screw channel, H is the depth of the screw channel, V_bz_ = π D_b_ N cos φ is the z-component of the peripheral velocity of the barrel, D_b_ is the diameter of the barrel, *n* is the rotational speed of the screw, φ is the helix angle of the screw, Δp__ch_ is the pressure change, L_p_ch_ is the screw length of the pressure change, m is the coefficient of consistency, and *n* is the power-law exponent.

## 5. Simulations

Simulations were made in the range of experiment data. The material data were taken from the literature [35]. The viscous flow properties (Figure 2) were modeled using the rheological equation of Klein [49] in the form of

(7) $$\ln\eta \;=\;A_{0}\;+\;A_{1}\ln\overset{\cdot }{\gamma }\;+\;A_{12}\ln\overset{\cdot }{\gamma }T\;+\;A_{2}T\;+\;A_{22}T^{2}$$

where η is the viscosity, Pa·s, $\overset{\cdot }{\gamma }$ is the shear rate, s^−1^, T is the temperature, °C, and A_0_, A_1_, A_11_, A_12_, A_2_, A_22_ are the Klein model parameters: for WPC of WF/PP = 20/80 (A_0_ = 12.971, A_1_ = −1.3878, A_11_ = −0.026553, A_12_ = 0.0057936, A_2_ = 0, A_22_ = −0.00014641), and for WPC of WF/PP = 40/60 (A_0_ = 12.771, A_1_ = −1.2174, A_11_ = −0.013446, A_12_ = 0.0037079, and A_2_ = 0, A_22_ = −0.00010186).

**Figure 11:**
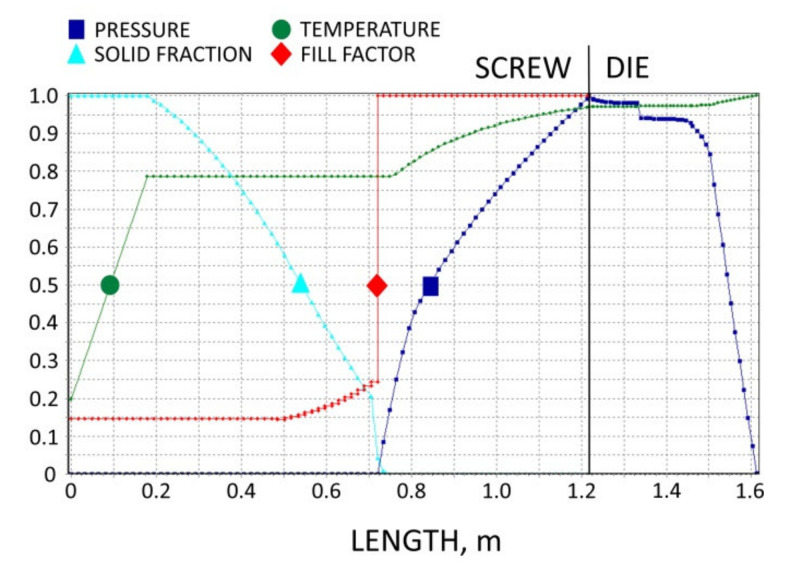
Overall extrusion process characteristics: WPC of WF/PP = 40/60, *n* = 30 rpm.

**Figure 12:**
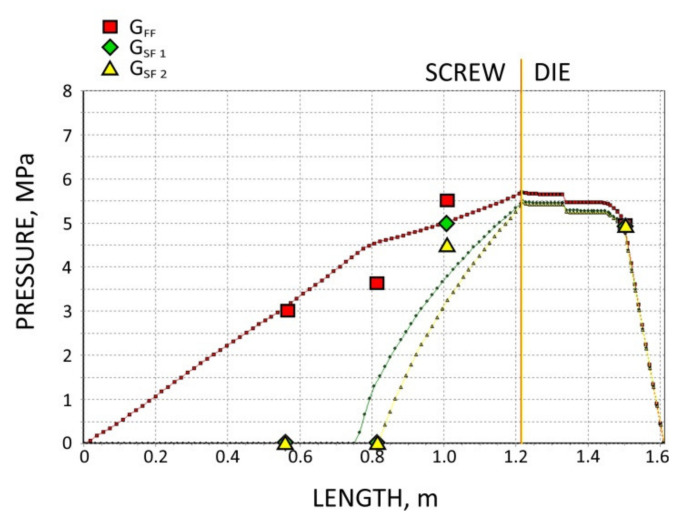
Validation of pressure computation: WPC of WF/PP = 20/80, *n* = 30 rpm.

**Figure 13:**
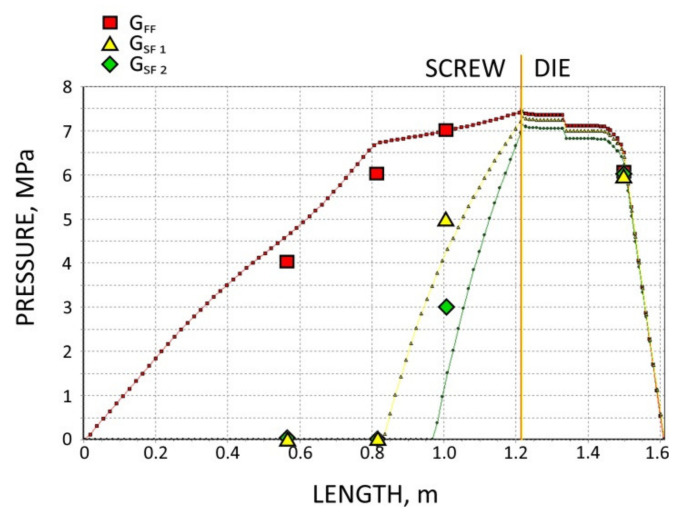
Validation of pressure computation: WPC of WF/PP = 20/80, *n* = 70 rpm.

**Figure 14:**
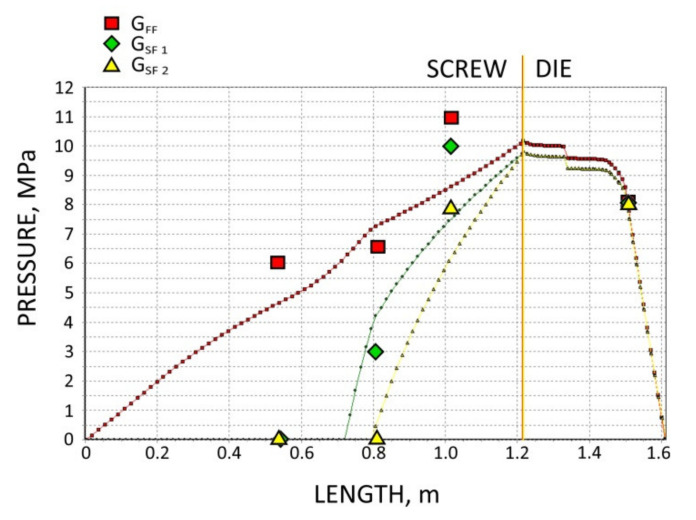
Validation of pressure computation: WPC of WF/PP = 40/60, *n* = 30 rpm.

**Figure 15:**
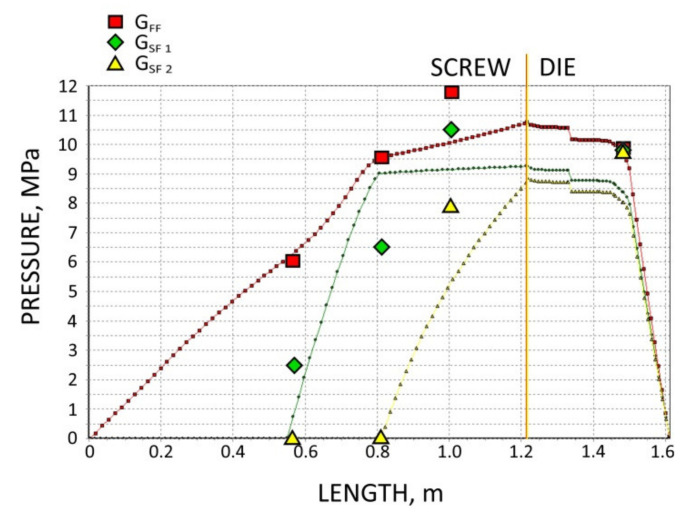
Validation of pressure computation: WPC of WF/PP = 40/60, *n* = 70 rpm.

The results of the simulation are presented in Figure 11, Figure 12, Figure 13, Figure 14 and Figure 15. The overall extrusion process characteristics are depicted in Figure 11. The dimensionless characteristics are composed of important process parameters: the pressure (P) and temperature (T) distributions, the melting (fusion) profile, i.e., the solid fraction (SF), and the screw filling profile, i.e., the fill factor (FF) which defines the extent of filling of the screw. The solid fraction (SF) is understood as a ratio of the solid polymer volume to the total polymer volume. The fill factor (FF) is understood as a ratio of the polymer volume to the incremental volume of the screw channel.

At the beginning of the screw, the material gets hot until the polymer fusion temperature is reached, and the solid fraction (SF) is equal to unity, SF = 1 (blue line). When the polymer fusion temperature is reached (green line), the fusion starts, and the solid faction (SF) reduces, SF < 1. When the screw channel gets fully filled (red line), the fill factor (FF) is equal to unity, FF = 1, and the second mechanism of fusion occurs. Finally, when the polymer is completely fused, the solid fraction (SF) falls to zero, SF = 0. There is no pressure development in the partially filled region (navy line) where the fill factor (FF) is lower than the unity, FF < 1. However, when the screw channel gets fully filled where the fill factor (FF) is equal to unity, FF = 1, the pressure is developed (navy line). The pressure rises in the screw and drops in the die. From the principle, the pressure increase in the screw is equal to the pressure decrease in the die.

In the study, the extrusion process was simulated for both composites (WF/PP = 20/80 and WF/PP = 40/60), at two screw speeds *n* = 30 rpm and *n* = 70 rpm, and at the flood/starve fed conditions, each time at two levels of starving (G_SF 1_ > G _SF 2_). The pressure computations were validated by experimental data.

The results of validation are presented in Figure 12, Figure 13, Figure 14 and Figure 15. The predictions were quite good, especially for the lower (20%) content of wood fiber (WF) which is seen in Figure 12 and Figure 13. For the higher (40%) content of wood fiber (WF), some discrepancies in a few experimental points were observed between the simulation and experiment, which is seen in Figure 14 and Figure 15. The pressure in the die was correctly predicted.

Wood plastic composites (WPC) are wall-slipping materials that exhibit yield stress, and these properties were not considered in this study. Thus, this might be the possible cause of discrepancies between simulations and experimentations. Another possible reason might be the effect of the elongational viscosity (important in the conical die) which may occur at the low shear/elongation rates, and which was also not included into the model. These were recently discussed in the literature [50].

An impact of these properties on the process flow, i.e., wall-slipping and yield stress, which are not included into the model, increases with an increase in the content of wood filler (WF) content. Moreover, for higher (WF) content, the fusion mechanism may be closer to the one-dimensional model which is observed for the composites of more than 50% (WF) content [35].

## 6. Conclusions

Experimental research has shown that the flow and fusion of the polypropylene (PP) based wood composites (WPC) in the starve fed extrusion are considerably different from those of the neat polypropylene (PP).

It was seen that in the initial part of the screw, the polymer thickens gradually, and it is generally fused in the whole mass, by conduction primarily as in the case of the extrusion of neat polymers. On the other hand, in the area of the fully filled screw, the classical Tadmor fusion mechanism appears with a melt pool at the active flight of the screw, which is different compared to the fusion mechanism of neat polymers at starve fed extrusion, where dispersed fusion is observed.

Based on the experimentation, the fusion mechanisms for starve fed extrusion of wood plastic composites (WPC) have been proposed. In the partially filled section, fusion by conduction is postulated, while in the fully filled section, the classical Tadmor mechanism is proposed.

Using these fusion models, the composite computer model of the starve fed single screw extrusion of wood plastic composites (WPC) has been built, which predicts the pressure and temperature distributions, the fusion rate, and the degree of screw filling. To build the model, the specific forward/backward computation procedure has been developed, which consists in computing “forward” the fusion profile, and ”backward” the pressure and screw filling profiles. The temperature profile in the fusion section is computed “forward”, while it is computed “backward” in the metering section. This procedure makes it possible to solve the crucial problem of modeling the starve fed extrusion process, which is to find the location of the point where the screw is fully filled. From this point, the pressure is developed and the fusion mechanism changes. The location of this point varies at each iteration of computation and it is found by searching the convergence of the computed polymer melt temperature and the polymer fusion temperature at the point where the fusion finishes.

The model has been successfully validated experimentally by pressure measurements. However, for the higher (40%) content of wood fiber (WF) some discrepancies were observed between the simulation and experiment.

The possible causes of these discrepancies might be the specific properties of wood plastic composites (WPC), such as wall-slipping and yield stress, which were not included into the model. Thus, including these into the model could be suggested, as well as further experimentation.

It can be concluded that starve fed extrusion, which was discussed first in the eighties and then was the subject of very little research, was finally successfully modeled in recent years, and is worthy of intensive experimental and modeling research, especially with regard to the demanding processing tasks related to the processing of advanced materials, such as filled polymers, polymer blends, or polymer composites.

## Data Availability

The data presented in this study are available on request from the corresponding author.
